# Development and Preliminary Italian Validation of the Emergency Response and Psychological Adjustment Scale

**DOI:** 10.3389/fpsyg.2021.687514

**Published:** 2021-08-05

**Authors:** Pierluigi Diotaiuti, Giuseppe Valente, Stefania Mancone

**Affiliations:** Department of Human Sciences, Society and Health, University of Cassino and Southern Lazio, Cassino, Italy

**Keywords:** emergency response, disruption adjustment, disaster recovery, mental health, confirmatory analysis, concurrent validity, measurement invariance

## Abstract

Evaluating the personal adaptation response to the emergency situations is very important for the prevention of mental distress, for the activation of network and community synergies and for the planning and implementation of appropriate psycho-social interventions. So far there are no short tools for the overall assessment of cognitive, emotional and behavioral responses of psychological adaptation to the emergency in the psychometric panorama. The Emergency Response and Psychological Adjustment Scale (ERPAS) was administered to a sample of 1,088 participants, while the concurrent validity was tested through a second administration to 600 participants along with the GSE (*Generalized Self-Efficacy Scale*) and the BDI-II (*Beck Depression Inventory-II*). Confirmatory factor analysis bore out a five-factor solution (including 18 items) with good fit indices of adaptation to data, χ^2^/*df* = 1.440, RMSEA = 0.028, RMSEA 90% CI = 0.018–0.038, GFI = 0.996, AGFI = 0.959, CFI = 0.982, and NFI = 0.944. Evidence of convergent validity was provided by the significant correlations with variables such as cognitive and somatic depression, and perceived general self-efficacy. The analyses also showed a strong invariance across gender. The ERPAS tool prefigures application during the assessment in multiple emergency contexts (e.g. earthquakes, floods, pandemics, terrorist attacks, war events, major accidents, major fires). This validation study of the *ERPAS* has shown that this version is a reliable and valid measurement for assessing people's modes of personal response (cognitive, emotional, behavioral) in emergency contexts.

## Introduction

Psychological support actions in emergencies are usually aimed at reducing the initial stress caused by events and facilitating short-term adaptive functioning (IASC, 2007). Reducing the stress experience will, in turn, enable the activation of useful energy and behavior in emergencies and reduce, in the long term, the painful intensity of dramatic memories (Figueroa et al., 2010; Silgo, 2014; Sepeng and Makhado, 2019).

Being able to function adaptively to the special situation can become important in two ways: first, it will be easier to put in place help and protection measures, follow the rescuers’ instructions and make correct and context-appropriate choices. Secondly, it will uproot the memory of an experience in which, despite everything, effective behavior has taken place (Vernberg et al., 2008). This leads to a defense of the self-image and sense of self-esteem and self-efficacy, so important to reinforce resilient strategies (Wolfenstein, 1998; Monteil et al., 2020).

An important strategy is to increase the perception of security, facilitating the ability of individuals to control their environment, themselves, relationships, and ongoing events. Increasing the sense of control in the face of events that have taken over facilitates the early restoration of confidence in one's ability to protect oneself (Zaumseil et al., 2013).

The majority of people in the face of an adverse event react adaptively and a minority of people experience negative changes that can be classified into three categories with areas of overlap: psychopathological and psychiatric disorders, psychological distress that cannot be classified in any disorder and health-relevant behaviors (Benedek et al., 2007).

Post-traumatic disorders are the most studied but not the most relevant after a disaster, indeed the most frequent diagnostic categories seem to be anxiety disorders, depression, and substance use disorders (Rubonis and Bickman, 1991; Stein et al., 2000; Johansson and Nadeau, 2006).

More often there are distress reactions not specifically attributable to classified mental disorders. These are symptoms of psychological distress that manifest themselves through the experience of negative emotions such as anger, sadness, fear, anxiety, irritability, nervousness, or the alteration of interpersonal interactions such as tension, social withdrawal, conflicts in the family, or the reduction of working capacity with poor concentration (Saadatian-Elahi et al., 2010; Blakey and Abramowitz, 2017; Jalloh et al., 2018; Huang and Zhao, 2020).

The third area concerns health risk behaviors. Some surveys show an increase in the use of alcohol or cannabis, an increase in sexual risk behaviors, cigarette consumption, or a lower propensity to quit smoking (Schiff et al., 2007; Peltzer and Pengpid, 2014, 2018; López-Bueno et al., 2020).

As far as the duration of the effects is concerned, it is maximum within the first year and then gradually decreases (Norris and Elrod, 2006). Among the risk factors Rubonis and Bickman (1991) highlighted the centrality of the characteristics of the disaster: as the number of deaths increases, so do the rates of psychopathology; technological disasters are associated with higher rates of psychopathology than natural disasters; interpersonal traumatic events such as violence have a greater impact on mental health than accidental or natural ones.

Brewin et al. (2000) reported that personal and social factors before and after a trauma have a significant effect on the risk of developing PTSD. Before a trauma, personal risk factors are mainly mental health problems; after a trauma, risk factors are poor social support and exposure to further stressful events (Ozer et al., 2003).

However, the protective effect of social support depends on the nature of the adverse event: in the case of visibly traumatic events, considered as such by the community where the survivor appears to be a hero, victims have more access to social support and benefit more from it, while in the case of ambiguous, private events, characterized by social stigma or characterized by impotence or shame (such as rape, child abuse, accidents in which the victim is guilty), it is much less likely that a social support network is activated (Charuvastra and Cloitre, 2008; Mafune et al., 2019; Nguyen-Trung et al., 2020; Sanandres et al., 2020).

Beyond the clinical repercussions, exposure to disaster affects relational and family life and life transitions in general. According to the classical perspective of stress, a stressful event influences by increasing tension, irritability, and worsening the quality of the relationship. A disaster, as a life-threatening event, can be a catalyst for people in making important decisions regarding their emotional and family path (Cohan and Cole, 2002; Reid and Reczek, 2011; Lowe et al., 2012; Shin et al., 2018; Prime et al., 2020). A life-threatening event shakes the basic assumptions about safety, predictability, justice and the comprehensibility of what happened, thus revealing the discrepancy between the beliefs in the world as safe and predictable and the reality of danger and randomness and necessarily motivating one to review old patterns in order to define new ones. People are therefore urged to review the priorities and goals of their lives and translate this reconstruction into action (Nakonezny et al., 2004; Allen, 2006; Riffle et al., 2020).

Within the so-called positive psychology approach, a strand that has begun to take an interest in the positive aspects of trauma gives way to the idea that a crisis can be transformed into growth. Tedeschi and Calhoun (1996) introduced the expression “post-traumatic growth” by reasoning on the idea that after a trauma the individual can undergo positive changes in three areas: self-perception, interpersonal relationships, and life philosophy.

First of all, self-perception can change for the better when people no longer feel like victims or survivors, but people who have overcome a difficult event and who are now living and not simply surviving. Considering oneself in this way can foster personal growth, for example in terms of increased self-confidence and a better assessment of one's ability to cope with difficulties. At the same time, people can become more aware of their frailty and vulnerability (Degortes et al., 2003; Sattler et al., 2014).

Secondly, after a trauma there is a positive change in interpersonal relationships: the person enters into new relationships, strengthens old ones, breaks unsuccessful ones, and feels more intense emotional closeness with someone important (Lahav et al., 2017). The sense of vulnerability experienced during the event can also increase the expressions of emotions, the acceptance of help, empathy or altruism for others who live similar situations and strengthen their self-esteem.

Thirdly, positive change can affect the philosophy of life. Those who have seen their lives so deeply threatened learn to appreciate it more, desire to live more fully and intensely by making more conscious and courageous choices or by dedicating more effort to change what needs to be changed (Prati and Pietrantoni, 2006; Maltais et al., 2020).

Evaluating the personal adaptation response to the emergency situations is very important for the prevention of mental distress, for the activation of network and community synergies and for the planning and implementation of appropriate psycho-social interventions. So far there are no specific tools for the overall assessment of cognitive, emotional and behavioral responses of psychological adaptation to the emergency in the psychometric landscape. Although several clinical guiding tools are available for diagnosing posttraumatic disorder, such as the PDS (*Post-Traumatic Stress Diagnostic Scale*, Foa, 1995), the DES (*Dissociative Experience Scale*, Bernstein and Putnam, 1986; Carlson and Putnam, 1993), the DTS (*Davidson Trauma Scale*, Davidson et al., 1997), the CAPS-5 (*Clinical-Administered PTSD Scale*, Weathers et al., 2013), measurement of the response and adaptive behaviors of individuals who are experiencing emergency situations does not have a corresponding tool to date. In 2020, Zsido et al. proposed the *Emergency Reaction Questionnaire*, a recent 30-item, four-factor instrument designed to predict operator readiness in emergency situations (fighter pilots, firefighters, and ambulance crew); the purpose of this tool is to predict mostly one's reaction in an emergency and help decide whether the person will be able to start immediate remedial actions and do it in an organized way or will this person start to panic and block or set back others’ actions.

In this study, we thought it appropriate to develop and present a new instrument capable of assessing in the general population not only the immediate responses (emotional and cognitive) to the current critical condition, but above all the presence and development of behaviors and thoughts more functional to the path of adaptation and overcoming the critical condition that is being faced. In addition to the need (defensive) to be able to contain and manage the strong fears, frustrations, concerns that the unusual overwhelming condition activates, it is important to assess the acquisition of progressive awareness and mature acceptance of the situation, in order to activate a process of learning from the experience: the person becomes aware of the situation, begins to think about how to behave and adapt in this new condition, identifies a daily goal, begins to plan future goals to commensurate with the new situation, and recognizes and is aware of his emotions. Our theoretical reference of this path of functional and conscious adaptation is the PTG model previously mentioned (*Posttraumatic Growth*, Tedeschi and Calhoun, 2004). In the adaptation process the person also understands that in addition to taking care of himself, he/she can also share some of his talents with those in need, he/she looks for ways to be useful to others while discovering a new way of adapting to change and he/she becomes empathetic with himself. In this scheme it is possible to identify an opportunity for the growth of the person, who does not stiffen by withdrawing in his anxieties, but externalizes attention with prosocial and network initiatives (Yue and Yang, 2021). Adaptive and supportive modes could contribute significantly to nourish the person's sense of self-efficacy and limit personal vulnerability to stress (see Saccinto et al., 2013; Diotaiuti et al., 2021b). Recently, in their study Hou et al. (2020) stressed the protective role of self-efficacy in limiting the symptoms of post-traumatic stress disorder and the perception of fatigue in health care workers.

There are numerous studies in the literature that point out that extreme and highly stressful events can have a different impact on men and women, conditioning their adaptation strategies. In Ziabari and Treur (2018) we find an interesting reference review, where it is reported that from the field of epidemiological research it has emerged that females are much more likely to get anxiety disorders than males (Arrindell and Luteijn, 2000); while a neurological perspective would have shown that females have a weaker Hypothalamic-pituitary-adrenal axis (HPAA) and autonomic reactivity than males (Kajantie and Phillips, 2006). In McClure et al. (2004) it is noticed that females have more activity in their cortex and orbitofrontal cortex in facing threats than males. Kendler et al. (1992) found out that when females get stressed, the level of oxytocin will increase and it improves their tendency of accompanying with others. In Craske (2003) it is contemplated that females have stronger feelings of worry whenever they face threats. Already Endler et al. (1962) reported that females get a higher score on STAI (which is a cognitive and affective describer of anxiety) than males. Referring to anxiety, Stewart et al. (1997) claimed that females have much more fear of physical outcomes of anxiety, while in Foot and Koszycki (2004) it is reported that males have much more fear of the social outcomes of anxiety. Wood and Eagly (2002) described that propensity to consider vague conditions as a threatening situation is an adaptive method for females to maintain the safety of themselves and their offspring. In McLean and Anderson (2009) it is declared that males are more into individual problem solving and as such they tend to focus on coping with emotion and anxiety in ways different from females. An updated gender perspective in disaster studies is copiously offered by the Reference Guide edited by the Center for Gender and Disaster (Centre for Gender and Disaster, 2020) and part of a project aimed at integrating gender studies in disaster risk reduction. In light of this evidence, the tool ERPAS (Emergency Response and Psychological Adjustment Scale) proposed in our study is being subjected to gender invariance analysis, aiming to explore whether the measurement of reaction and adaptation to the emergencies through the tool proposed would also result invariant as a function of gender.

Convergent validity was assessed using two tools widely used in previous research on the psychological impact and monitoring of individual responses to stress, trauma, bereavement, disaster, and situations with strong general emotional involvement: BDI-2 (Beck Depression Inventory) and GSE (the General Self-Efficacy Scale). With reference to previous literature, a positive association of BDI-2 with the factors of Agitation and Worry was hypothesized, negative associations with the other adaptation factors (Awereness, Prosociality, and Perceived Self-Efficacy); while for GSE, an inverse relationship with the reactions of Agitation and Worry was hypothesized, and a positive association with the three adaptation factors of ERPAS.

## Materials and Methods

### Development of the Emergency Response and Psychological Adjustment Scale: Item Generation and Content Validity

In order to develop and provide evidence for the content validity of a pool of items that could assess people's response and psychological adjustment to the emergency, have been preliminarily involved both common individuals aged between 18 and 65 years and professionals experienced in the field of emergencies. Precisely, a group of 21 people (11 females and 10 males) representing the age groups 18–40, 41–59, and >60 and a group of 10 experts (two Civil Protection officials, two officers of the Carabinieri and Police, two officers of the Fire Department, two Army officers, two emergency psychologists). The criterion for inclusion and involvement in this second group was, in addition to a seniority of service of more than 15 years, to have served on active duty in the last 3 years to at least two of the following types of emergencies (earthquake, floods, landslides, fires, explosions, firefighting, war events, epidemics, rail, or air accidents).

The items, referring to responses and psychological adjustment to the emergency were developed over several stages. At the first stage, the relevant psychological literature on common behavior and psychological reactions in emergency situations (e.g., Williams and Drury, 2009; Grimm et al., 2014; Cheng et al., 2019) was used as a reference in the development of the emergency-specific items. At the second stage, we performed a semi-structured interview with each expert (30 min), one focus group (on line) with the whole expert group (60 min), and two focus groups (90–110 min) with the group of common people. The following domains were identified: (1) Worry (i.e., basic necessities, money availability, extensive consequences of the crisis, questioning of future projects; 10 items), (2) Agitation (i.e., anxiety, stress, fears, irritability; 8 items), (3) Awareness (i.e., learning, focusing on the present, adjustment to changes, daily purpose identification; planning for the future; 10 items), (4) Prosociality (i.e., solidarity, making oneself available to others, aiding others in need, instilling hope in those around; empathizing with others; 8 items); (5) Self-Efficacy (i.e., feeling able to face unexpected events, relying on one's own abilities in emergencies, being able to remain calm in facing difficulties; 9 items). At the third stage, common people who participated in stage two assessed the relevance of each item in the context of emergency using a dichotomous scale (1 = applicable, 0 = inapplicable). Items that were deemed inapplicable by one third (33%) or more of the people were eliminated. Applicable items that were rated below 5 were considered problematic (1 = not at all clear to 7 = extremely clear); participants were encouraged to suggest alternative wordings for these problematic items. At the final stage, a reduced pool of items was sent via email to the experts. Two steps were taken in this stage. Firstly, the 10 experts were asked to rate the representativeness of each item with regard to the concept of emergency response and psychological adjustment, using a 4-point response scale from 1 (not relevant) to 4 (highly relevant). Secondly, five of the ten experts were again asked to rate the representation of the revised items using the same 4-point response scale (see Polit et al., 2007). The item-level content validity index (I-CVI; Lynn, 1986; Polit et al., 2007) was calculated for each item by dividing the number of experts who rated the item as a quite relevant or highly relevant (rating 3 and 4) by the total number of experts who provided ratings. When an expert panel consists of six or more reviewers, I-CVIs over the 0.78 criteria are 219 considered to be excellent (Lynn, 1986). The scale-level content validity index (S-CVI/Ave) was calculated by averaging all the I-CVIs; an S-CVI/Ave over 0.90 is considered to be satisfactory (Polit et al., 2007). Initially, 35 items were generated and another 10 items were suggested by experts, which formed a pool of 45 items. Based on the first group evaluations, 12 items were deemed inapplicable in the emergency context and were thus eliminated (e.g., “I am concerned about my psychological well-being”), whereas seven items were modified to improve their clarity and broaden their applicability across emergencies (e.g., “I am sure that I can deal effectively with unexpected events”). Of the remaining 33 items, three items that displayed a CVI of 0.70 (7/10) or below were deleted. Minor modifications were made to the wording of two items. This process resulted in a pool of 30 items, with a satisfactory S-CVI/Ave of 0.96. The instruction identified for the items asked the person to assess how the situations listed were consistent with their current experience. The items were rated on a 5-point scale ranging from 1 = not at all to 5 = extremely. Table 1 below presents the 30 selected items.

**Table 1:** Selected items.

1	I am sure that I can deal effectively with unexpected events.
2	I remain calm in facing difficulties because I can rely on my abilities.
3	It doesn't matter what can happen because I feel able to handle it.
4	I can always solve difficulties if I try hard enough.
5	I am worried about being without basic necessities (food, medicine, clothes).
6	I am concerned about a possible economic crisis linked to the situation.
7	I am concerned about my financial resources.
8	I'm worried about my future plans.
9	I am concerned that the difficulties will last for a longer period of time than expected.
10	I am concerned about my psychological well-being.
11	I am concerned about the psychological well-being of my family members.
12	I am upset about this change in habits.
13	I am feeling anxious at this time.
14	My stress level has increased.
15	I hoard food, toilet paper, and medicine even if I don't need it.
16	I get frequently irritated.
17	I let myself be infected by the fear and anger of others.
18	I often get irritated.
19	I become aware of the situation and think about how to behave.
20	I live in the present and focus on the future.
21	I look for a way to adapt to new changes.
22	I am trying to find a purpose in the day.
23	I limit excesses that could hurt me, from food to news.
24	I let go of what I can't control.
25	I identify my emotions.
26	I recognize that we are all trying to do our best.
27	I think about others and look for ways to help them.
28	I make myself available to anyone who is in need.
29	I maintain a positive emotional state and instill hope in those around me.
30	I am empathetic to others.

In order to preliminarily test the hypothetical five-factor structure corresponding to five main content domains that emerged from the literature consultation and derived from the item generation and content validity process, a pilot administration of these 30 items was carried out involving 60 university students attending the psychology course, who freely took part in this first pilot analysis. EFA with Maximum Likelihood and Promax rotation preliminarily confirmed the hypothesis of a five-factor model with 44% cumulative variance, and fit indices RMSEA = 0.056; RMSEA 90% CI = 0.05–0.059, TLI = 0.873. EFA with Maximum Likelihood and promax rotation preliminarily confirmed the hypothesis of a five-factor model with 44% cumulative variance, and fit indices RMSEA = 0.056; RMSEA 90% CI = 0.05–0.059, TLI = 0.873. Therefore an extended sample was subsequently administered.

### Participants and Administration Procedure

**Figure 1:**
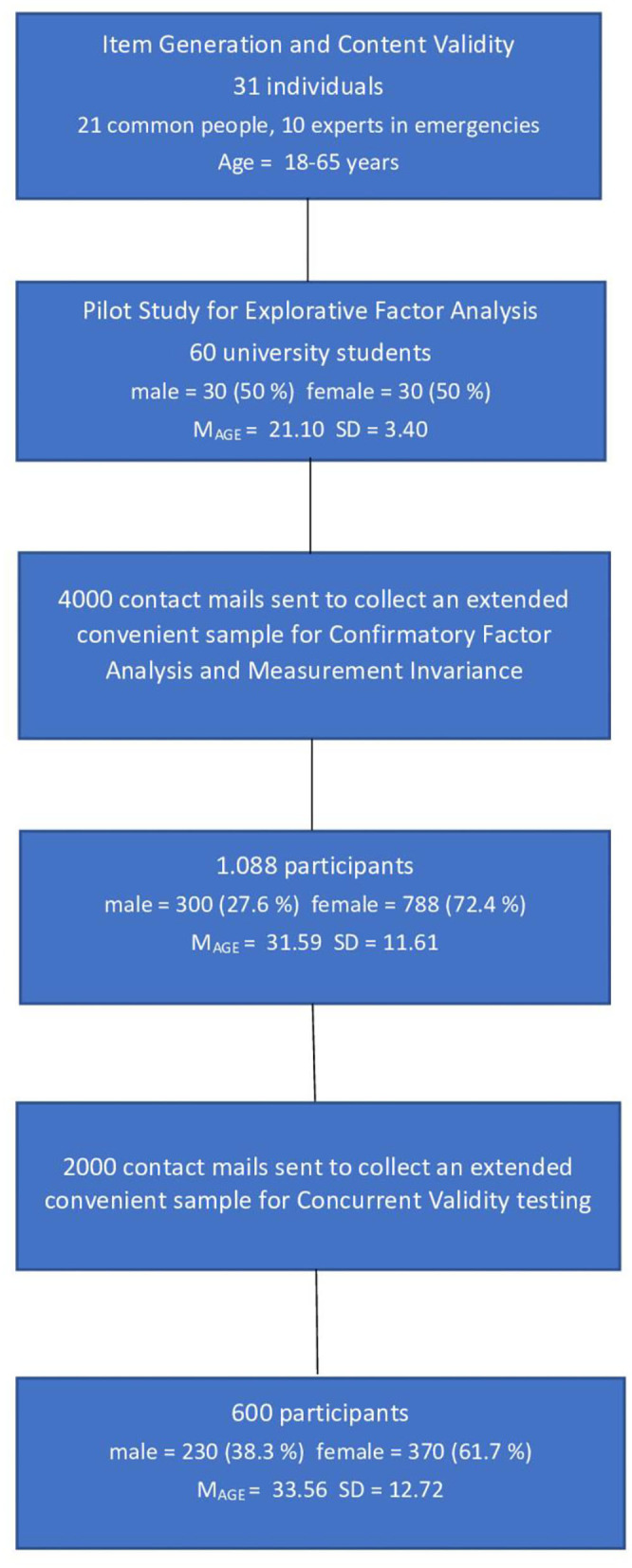
Consort Diagram.

For the purposes of Confirmatory Factorial Analysis (CFA) analysis to test the psychometric adequacy of the *ERPAS* instrument, the scale was administered during the emergency COVID-19 lockdown period to an Italian sample of 1,088 participants, 300 (27.6%) males and 788 (72.4%) females with an average age of 31.59 and *SD* = 11.61. The data were collected through the administration of the questionnaire to a convenience sample of residents in the area of central/southern Italy. Students of the local university were involved in a representative proportion (at least 30%) of the three regions of major provenance (Lazio, Campania, Molise). Each student was asked to involve (by forwarding an email requesting participation) at least three family members and/or friends who were each in the age range 18–38; 39–59; >60. Therefore, the exclusion criteria were an age below 18 years and an age above 65 years. In the first case, the reason for exclusion was related to the problem of parental consent, in the second case, the telematic administration of psychological assessment tools to elderly individuals was deemed inappropriate. For the purposes of the study, the university administration granted access to the database of email contacts of enrolled students; the database also contained information on the residence of these students. Participants have therefore received an email inviting them to freely join the research by answering an online questionnaire. Four thousand emails were sent out (March 4), extracting them from a list of approximately 9,000 contacts. Data collection began on 4 March and ended on 30 April 2020. Participants were assured anonymity and the use of data in aggregate form for research purposes only. It was specified that they would not receive remuneration for their participation and if they had any doubts or problems they could contact the study representative directly. The average completion time was about 15 min. Tools administration took place upon the release and signing of the form for an informed consent of participation in accordance with the Declaration of Helsinki. The concurrent validity was tested through a second sample of participants consisting of 600 individuals (230 males 38.3% and 370 females 61.7%) with an average age of 33.56 and *SD* = 12.72. Everyone accepted voluntarily to participate in the study after being informed of its objectives and they all supplied an adequate compilation of the instrument. They were also informed of the anonymity of the test and the fact that it was designed for research purposes only. The protocol was approved by the local university Institutional Review Board. The following Consort Diagram (Figure 1) shows the whole participants enrollment and flow of the study.

### Measures

#### The Generalized Self-Efficacy Scale

*The Generalized Self-Efficacy Scale* (GSES: Schwarzer and Jerusalem, 1995; *it. val*. Sibilia et al., 1985): consisted of 10 items on a 4-point Likert scale (reliability for this study: alpha = 0.87; omega = 0.88) ranging from 1 (completely false) to four intervals (completely true) and was used to assess the general sense of perceived self-efficacy in order to predict coping with daily hassles as well as adaptation after experiencing all kinds of stressful life events. The scale refers to the personal agency, i.e. the belief that individual actions are responsible for successful results.

#### Beck Depression Inventory-II

*Beck Depression Inventory-II* (BDI-II, Beck et al., 1996; *it. val*. Ghisi et al., 2006): it is widely used by clinicians in screening and tracking depression symptoms and consists of 21 items that are summed in order to create a composite score of depression. Examples of these items include questions regarding changes in sleep patterns, difficulty concentrating, sadness, self-dislike, crying, loss of energy, and suicidal thoughts, in which four response options are presented on a scale of 0–3. For example, to measure pessimism (item 2) the response options used range from “I am not particularly discouraged about the future” (score of 0) to “the future is hopeless and things cannot improve” (score of 3). These items were designed to capture the depression as defined by the *Diagnostic and Statistical Manual of Mental Disorders, Fourth edition* (American Psychiatric Association, 2013) (reliability for this study: alpha = 0.90; omega = 0.92).

### Statistical Analysis

The sample size was based on the ability to verify an adequate fit of *ERPAS* starting with a version that included a five-factor model with 30 manifest variables. Using the root-mean-square error of approximation (RMSEA) as the measure of model fit, a minimum of 300 participants provides a 90% power level to test RMSEA **≤** 0.05 when RMSEA = 0.08, using a 0.05 significance level (MacCallum et al., 1996). The main statistical analyses carried out were the following: verification of the assumptions of univariate and multivariate normality; CFA; assessment of internal consistency through Cronbach's alpha coefficient and McDonalds ω evaluation of significance of correlation coefficients to test concurrent validity of the tool. Statistical analyses were performed using the packages SPSS version 22, JASP 0.12.2, and IBM Amos Graphics 18.

To test the adequacy of the model the following 10 indices were considered: (1) chi square; (2) the relationship between the chi-square value and the degrees of freedom (χ^2^/df, values between 1 and 3 are considered acceptable); (3) GFI (*Goodness of Fit Index*), with values higher than 0.90 indicating an acceptable fit of the model, while a good fit with values higher than 0.95; (4) AGFI (*Adjusted Goodness of Fit Index*), with values higher than 0.90 indicating an acceptable fit of the model, while a good fit with values higher than 0.95; (5) RMSEA (*Root-Mean-Square Error of Approximation*), with values between 0.05 and 0.8 indicating an acceptable fit of the model, while a good fit with values lower than.05; (6) *p-value for the test of close fit*, with values between 0.50 and 1 indicating an acceptable fit of the model, while a good fit with values between 0.05 and 0.50; (7) CFI (*Comparative Fit Index*), and TLI (*Tucker-Lewis Index*), with values between 0.95 and 0.97 indicating an acceptable fit of the model, while a good fit with values between 0.97 and 1; (8) NFI (*Normed Fit Index*), with values between 0.90 and 0.95 indicating an acceptable fit of the model, while a good fit with values between 0.95 and 1 (Hu and Bentler, 1999; Byrne, 2001; Schermelleh-Engel et al., 2003; Barbaranelli and Ingoglia, 2013); (9) PNFI (*Parsimony Normed Fit Index*), with values between 0.50 and 0.60 indicating an acceptable fit of the model, while a good fit with values between 0.60 and 1; (10) PCFI (*Parsimony Comparative Fit Index*), with values between 0.50 and 0.60 indicating an acceptable fit of the model, while a good fit with values between 0.60 and 1 (Mulaik et al., 1989).

To study reliability, the Composite Reliability Index (CRI) and the Average Variance Extracted Index (AVEI) were used. Values above 0.70 for the AVEI are considered good, and values of 0.50 are considered acceptable. For the CRI, values above 0.70 are considered good (Raykov, 1997). All values outside this range were considered not acceptable.

Measurement invariance of the factorial structure of the *ERPAS* by gender was assessed. Three nested models with increasing degrees of restriction were tested: the base model assessed configural invariance and allowed free estimation of all the parameters for each group. The metric (weak) invariance model, nested in the configural model, added the restriction of invariant factor loadings among groups. The scalar (strong) invariance model, nested in the second model, added the intercept constraint of the invariant items among the comparison groups. Finally, we tested strict invariance by comparing the scalar model to a model that also constrains residuals to be equal across tested groups. Given that the Chi-square indices are sensitive to the sample size, we focused mainly on the comparison of the CFI, TLI, and RMSEA indices. We considered a variation of these indices higher than 0.01 as a criterion to rule out the invariance of the more restrictive model and accept the more parsimonious model (Cheung and Rensvold, 2002). When the strict invariance was verified, the group mean differences in latent variables were tested.

Concurrent validity was determined by comparing the correlations between the *Emergency Response and Psychological Adjustment Scale* factors and the factors that make up GSES and BDI-II. To measure concurrent validity, Pearson coefficients were computed.

## Results

Since the conclusion of the telematic questionnaire obliged participants to respond to all items and request fields, were not incomplete responses and missing data in the final matrix. The verification of the assumptions of univariate and multivariate normality has been conducted using the procedure for the standardization of the variables, erasing the outlier cases with values >3, then secondly, after calculating the Mahlanobis Distance, eliminating the multivariate outlier cases with *D*^2^ greater than the critical value, calculated by considering chi-square as the reference distribution (level *p* < 0.001) with *p* degrees of liberty equal to the number of variables (Barbaranelli, 2006). The calculation of the Mardia Index (average of the squares of the Malhanobis Distances) produced a coefficient (1,066.49) lower than the limit value (1,088). This selection of cases from the original matrix implied the elimination of 271 participants. Therefore, the rest of the validation procedure was carried out with 1,088 cases, 300 of which were males (27.6%) and 788 females (72.4%). The average age was 31.59 with *SD* = 11.61.

The evaluation of the metric properties of the scale was conducted through a confirming analysis (CFA) designed to test the goodness of a five-dimensional model. The averages, standard deviations, and corrected item-total correlation for the single items and those differentiated by gender are reported in Table 2.

**Table 2:** Averages and standard deviations differentiated by gender.

**Item**	**Sample (***N*****=** 1,088)**		**Males (***N*****=** 300)**		**Females (***N*****=** 788)**		**CITC**
	***Mean***	***SD***	***Mean***	***SD***	***Mean***	***SD***	
Item 1	3.53	1.03	3.63	1.05	3.49	1.02	0.271
Item 2	3.73	1.02	3.81	1.04	3.70	1.01	0.296
Item 3	3.41	1.06	3.51	0.98	3.37	1.09	0.263
Item 4	4.13	0.78	4.11	0.83	4.14	0.75	0.136
Item 5	2.55	1.27	2.28	1.15	2.66	1.30	0.386
Item 6	3.96	0.91	3.73	0.96	4.04	0.87	0.413
Item 7	3.36	1.10	3.13	1.10	3.45	1.09	0.401
Item 8	3.69	1.06	3.21	1.02	3.87	1.01	0.501
Item 9	3.48	1.08	3.26	1.04	3.56	1.08	0.413
Item 10	3.13	1.15	2.75	1.12	3.27	1.13	0.480
Item 11	3.50	1.06	3.16	1.04	3.62	1.04	0.532
Item 12	3.06	1.01	2.97	1.10	3.09	0.97	0.347
Item 13	2.90	1.15	2.47	0.99	3.07	1.16	0.372
Item 14	2.83	1.20	2.52	1.13	2.95	1.20	0.391
Item 15	1.74	0.91	1.57	0.78	1.81	0.95	0.235
Item 16	2.45	1.18	2.13	1.06	2.57	1.20	0.337
Item 17	1.62	0.79	1.42	0.68	1.69	0.81	0.277
Item 18	2.50	1.11	2.11	0.98	2.65	1.12	0.372
Item 19	3.61	0.84	3.43	0.85	3.68	0.83	0.320
Item 20	3.44	0.93	3.32	0.91	3.48	0.93	0.278
Item 21	3.63	0.83	3.46	0.85	3.70	0.81	0.275
Item 22	3.54	1.02	3.37	1.09	3.61	0.99	0.278
Item 23	2.90	1.06	2.73	1.07	2.96	1.05	0.226
Item 24	2.76	1.01	2.67	1.04	2.79	1.00	0.077
Item 25	3.20	0.98	3.00	1.02	3.27	0.95	0.292
Item 26	3.34	0.94	3.08	0.99	3.44	0.90	0.238
Item 27	3.15	0.91	2.83	0.85	3.27	0.90	0.391
Item 28	3.20	0.97	2.89	0.94	3.32	0.95	0.385
Item 29	3.23	0.97	3.23	0.84	3.23	1.01	0.115
Item 30	3.45	0.96	3.08	0.86	3.59	0.95	0.311

SD, standard deviation; CITC, corrected item-total correlation. ^*^p < 0.05; ^**^p < 0.005; ^***^p < 0.001.

**Figure 2:**
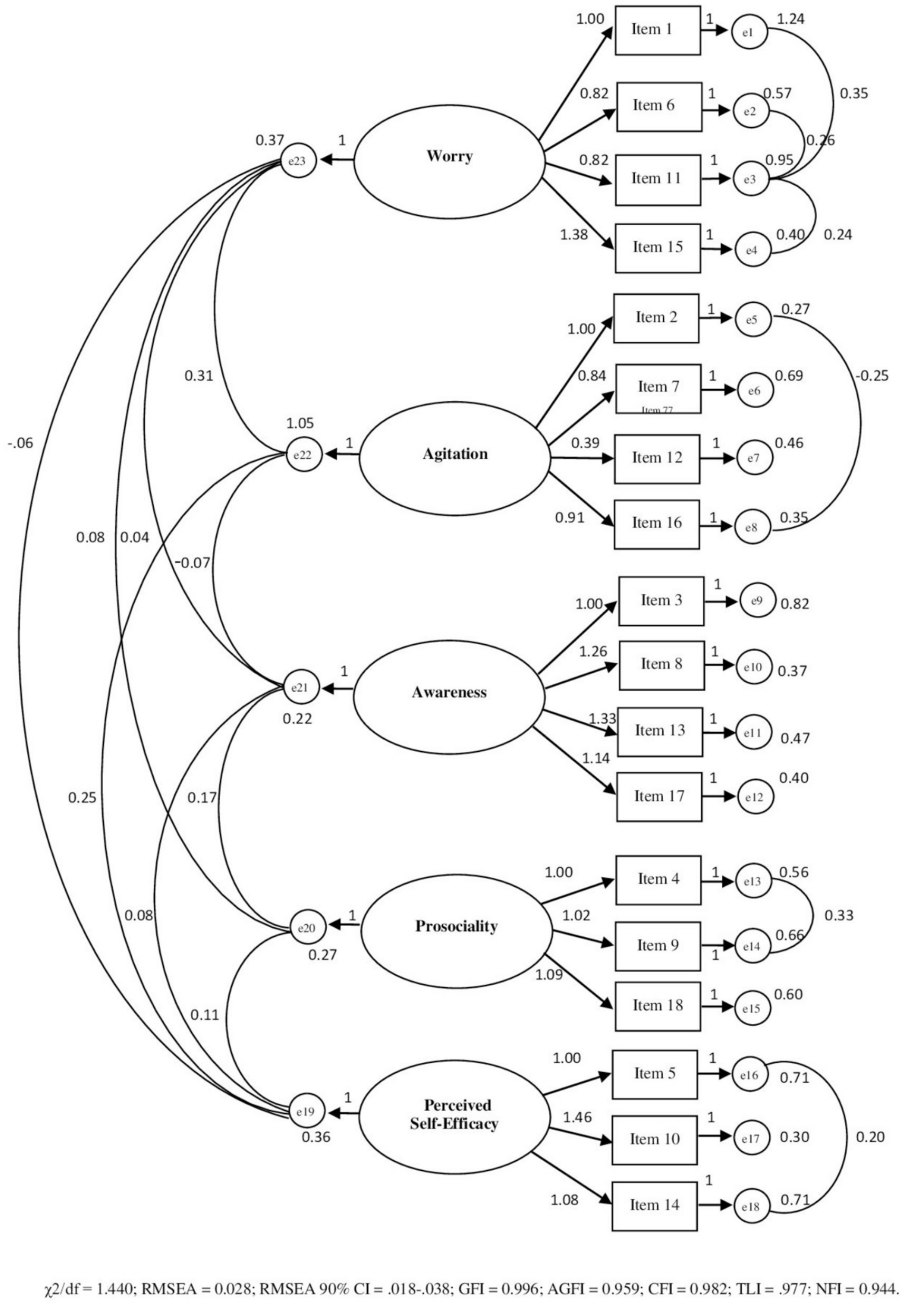
Path diagram of the confirmatory analysis concerning ERPAS (18 items).

The confirmatory factorial analysis with robust method and Maximum Likelihood estimator (see Figure 2) bore out that the model with five related factors and 18 items presented overall good indices of adaptation to data: χ^2^ = 171.423; χ^2^/*df* = 1.440; GFI = 0.996; AGFI = 0.959; CFI = 0.982; TLI = 0.977; RMSEA = 0.028; and RMSEA 90% CI [0.018–0.038]; p-close = 0.999; NFI = 0.944; PNFI = 734; PCFI = 0.770. The first factor measures *Worry* (four items); the second factor measures *Agitation* (four items); the third factor measures the *Awareness* (four items); the fourth factor measures the *Prosociality* (three items); the fifth factor measures the *Self-Efficacy* (three items).

Table 3 shows the model matrix with saturations on the five identified factors, McDonald's ω and Cronbach's Alpha values, Guttman Split-Half Coefficients, Corrected item/total correlations. All factorial loadings were statistically significant (*p* < 0.001) and ranged between 0.411 and 1.035. The AVEI (0.521) was accettable and the CRI (0.785) was good.

**Table 3:** Model Matrix (18 items).

	**Agitation**	**Worry**	**Awareness**	**Perceived self-efficacy**	**Prosociality**
Item 14	0.830	−0.014	−0.002	0.093	−0.028
Item 13	0.798	−0.008	0.007	−0.031	0.039
Item 18	0.718	0.015	−0.004	−0.041	−0.031
Item 17	0.480	0.038	−0.058	−0.025	0.019
Item 7	−0.126	0.928	−0.047	−0.012	−0.038
Item 6	0.014	0.599	0.060	−0.022	0.028
Item 8	0.243	0.570	0.074	−0.030	0.009
Item 5	0.045	0.542	−0.046	0.056	0.055
Item 19	0.059	−0.107	0.706	−0.076	0.097
Item 20	−0.033	0.011	0.683	0.041	−0.062
Item 21	−0.073	0.058	0.658	−0.013	−0.038
Item 22	−0.003	0.050	0.406	0.094	0.027
Item 3	0.062	−0.025	0.010	0.788	−0.075
Item 1	0.012	0.006	−0.016	0.697	0.016
Item 2	−0.094	0.025	0.037	0.639	0.090
Item 27	−0.012	−0.019	−0.045	0.000	0.979
Item 28	0.010	0.064	0.071	0.005	0.683
Item 30	0.045	−0.011	0.195	−0.012	0.392
α	0.80	0.76	0.70	0.76	0.71
ω	0.81	0.77	0.71	0.76	0.74
*λ6*	0.77	0.72	0.65	0.67	0.67
r*	0.50	0.45	0.38	0.51	0.45

Extraction Method: maximum likelihood. Rotation method: Promax with Kaiser normalization. Rotation converged in four iterations. Cumulative variance: 51.5 %. α, Cronbach's alpha; ω, McDonald's omega; λ6, Gutmann's lamda; r*, average inter-item correlation.

Furthermore, the measurement invariance of the factorial structure of the *ERPAS* by gender was assessed. Three nested models with increasing degrees of restriction were tested. Table 4 shows the goodness-of-fit indices of the multidimensional model by gender and nested models of invariance in ascending order of restriction level. Results showed that the *ERPAS* had strong invariance by gender and that the fit of the five-dimensional model for male and female was excellent.

**Table 4:** Measurement invariance by gender.

	**χ^**2**^**	***df***	**Δχ^**2**^**	**Δ** ***df***	**CFI**	**TLI**	**RMSEA**	**ΔCFI**	**ΔTLI**	**ΔRMSEA**	**AIC**	**BIC**	**ΔAIC**	**ΔBIC**
**Models in each group**														
Gender														
Female	112.489	102			0.994	0.992	0.016							
Male	126.782	102			0.968	0.957	0.042							
**Global models**														
Gender														
Configural	316.626*	238	–	–	0.972	0.964	0.035	–	–	–	24,895.51	25,342.60		
Metric	331.209*	251	14.583	13	0.971	0.965	0.034	−0.001	0.001	−0.001	24,884.09	25,275.30	−11.42	−67.3
Scalar	338.381*	264	7.172	13	0.973	0.969	0.032	0.002	0.004	−0.002	24,937.26	25,427.34	−53.17	152.04
Strict	384.099*	288	45.718	24	0.965	0.973	0.035	−0.008	0.004	0.003	24,934.98	25,321.89	−2.28	−105.45

*Df, degrees of freedom; χ^**2**^, Chi square; Δχ^**2**^, difference in Chi square; Δdf, difference in degrees of freedom; CFI, comparative fit index; TLI, Tucker-Lewis index; RMSEA, root mean square error of approximation; ΔCFI, difference in comparative fit index; ΔTLI, difference in Tucker-Lewis index; ΔRMSEA, difference in root mean square error of approximation; AIC, Akaike information criteria; BIC, Bayesian information criteria*.

* *p < 0.001*.

These results mean that the latent means can be compared by gender. The latent mean values were fixed to zero for females and, as could be seen in the following Table 5, males showed in this study lower latent mean values of Worry, Agitation, Awareness, and Perceived Self-Efficacy than females, while there were no significant differences in Prosociality.

**Table 5:** Group mean differences in latent variables.

**Variables**	**Factors**	**Mean**	***SE***	**CR**	***P***
Gender (male)*	F_1_	−0.79	0.12	−6.32	<0.001
	F_2_	−0.58	0.09	−6.43	<0.001
	F_3_	−0.41	0.12	−3.40	<0.001
	F_4_	0.15	0.11	1.40	0.161
	F_5_	−0.90	0.13	−6.77	<0.001

*SE, standard error; CR, critical ratio; F_1_, worry; F_2_, agitation; F_3_, awareness; F_4_, prosociality; F_5_, perceived self-efficacy*.

* *Reference variable is female*.

Concurrent validity was tested by examining the significance of correlation coefficients with *The* GSES (Schwarzer and Jerusalem, 1995; *it. val*. Sibilia et al., 1985) and BDI-II (Beck et al., 1996; *it. val*. Ghisi et al., 2006). A new sample was used for concurrent validity testing: 600 individuals (230 males 38.3% and 370 females 61.7%) with an average age of 33.56 and *SD* = 12.72. In relation to the results of these associations, three hypotheses have been formulated: (1) the higher the *Worry and Agitation*, the higher the total *Depression* would have been; (2) the higher the *Awareness, Prosociality and Perceived Self-efficacy*, the lower the *total Depression* and the higher the *General Self-Efficacy* would have been; (3) the higher the *Agitation*, the lower the *General Self-Efficacy* would have been. As shown in Table 6, the results have confirmed the assumed directions of correlation; therefore, the measure proved good convergent validity with the scales considered and consequently its usefulness in describing the main responses and the psychological adjustment of people in emergency conditions and also indirectly providing indications of their ability to deal with particularly critical and uncomfortable situations. McDonald's ω and Alpha coefficients for these convergent administrations ranged from 0.77 to 0.78 (*Worry*), from 0.79 to 0.80 (*Agitation*), from 0.70 to 0.72 (*Awareness*), from 0.84 to 0.85 (*Prosociality*) from 0.73 to 0.74 (*Perceived Self-Efficacy*), respectively.

**Table 6:** Correlations of the Emergency Response and Psychological Adjustment Scale (ERPAS) with the Generalized Self-Efficacy Scale (GSES) and the Beck Depression Inventory-II (BDI-II).

		**WO**	**AG**	**AW**	**PR**	**PSE**	**GSE**	**COG**	**SOM**	**DEPT**
***ERPAS***	WO	1								
	AG	0.337**	1							
	AW	0.072	−0.164**	1						
	PR	0.184**	−0.077	0.371**	1					
	PSE	0.041	−0.288**	0.210**	0.241**	1				
***BDI-II***	COG	0.164**	0.574**	−0.347**	−0.224**	−0.364**	1			
	SOM	0.157**	0.660**	−0.235**	−0.148*	−0.281**	0.768**	1		
	DEPT	0.169**	0.664**	−0.295**	−0.188**	−0.332**	0.909**	0.965**	1	
***GSE***	GSE	−0.014	−0.305**	0.271**	0.276**	0.901**	−0.433**	−0.297**	−0.370**	1

*WO, worry; AG, agitation; AW, awareness; PR, prosociality; PSE, perceived self-efficacy; GSE, generalized self-efficacy; COG, cognitive depression; SOM, somatic depression; DEPT, total depression*.

* *Correlation is significant at the 0.05 level (2-tailed)*.

** *Correlation is significant at the 0.01 level (2-tailed)*.

The following Table 7 reports the English and Italian versions of the ERPAS, and the grouping of the items on respective factors.

**Table 7:** Emergency Response and Psychological Adaptation Scale (ERPAS).

**English version**	**Italian version**
1. I am worried about being without basic necessities (food, medicine, clothes) (WO).	1. Sono preoccupato di rimanere senza beni di prima necessità (cibo, medicine, vestiti).
2. My stress level has increased (AG).	2. E’ aumentato il mio livello di stress.
3. I am trying to find a purpose in the day (AW).	3. Cerco di trovare uno scopo nella giornata.
4. I think about others and look for ways to help them (PR).	4. Penso agli altri e cerco modi per aiutarli.
5. I am sure that I can deal effectively with unexpected events (PSE).	5. Sono sicuro che posso affrontare efficacemente eventi inattesi.
6. I am concerned about a possible economic crisis linked to the situation (WO).	6. Sono preoccupato per una possibile crisi economica legata alla situazione.
7. I am feeling anxious at this time (AG).	7. In questo periodo mi sento ansioso.
8. I become aware of the situation and think about how to behave (AW).	8. Prendo coscienza della situazione e penso a come comportarmi.
9. I make myself available to anyone who is in need (PR)	9. Mi metto a disposizione di chi ha bisogno.
10. I remain calm in facing difficulties because I can rely on my abilities (PSE).	10. Resto calmo nell'affrontare le difficoltà perché posso far conto sulle mie capacità.
11. I am concerned about my financial resources (WO).	11. Sono preoccupato per le mie risorse economiche.
12. I let myself be infected by the fear and anger of others (AG)	12. Mi lascio contagiare dalla paura e dalla rabbia altrui.
13. I live in the present and focus on the future (AW).	13. Vivo nel presente e mi concentro sul futuro.
14. It doesn't matter what can happen because I feel able to handle it (PSE).	14. Non importa quello che può succedere perché mi sento in grado di gestirlo.
15. I'm worried about my future plans (WO).	15. Sono preoccupato per i miei progetti futuri.
16. I get frequently irritated (AG).	16. Mi irrito spesso.
17. I look for a way to adapt to new changes (AW).	17. Cerco un modo per adattarmi ai nuovi cambiamenti.
18. I'm empathetic to others (PR).	18. Sono empatico verso gli altri.

*WO, worry; AG, agitation; AW, awareness; PR, prosociality; PSE, perceived self-efficacy*.

Based on the distribution of the scores obtained from the normative sample, the cut-off criteria, differentiated by gender, have been identified, and reported in the following Table 8.

**Table 8:** Scoring directions of ERPAS.

**Factor**	**Low**	**Medium**	**High**	***M***	***SD***	***SE***	***SK***	***SE***	***KU***	***SE***
**Total sample (***N*****=** 1,088)**										
WO	4–12	13–15	16–20	13.57	3.33	0.14	0.06	0.10	−0.67	0.21
AG	4–8	9–11	12–20	9.86	3.40	0.15	0.47	0.10	−0.30	0.21
AW	4–13	14–15	16–20	14.22	2.64	0.11	−0.03	0.10	0.26	0.21
PR	2–6	7	8–10	6.35	1.72	0.07	0.28	0.10	0.02	0.21
PSE	3–10	11–12	13–15	10.67	2.55	0.11	−0.56	0.10	0.16	0.21
**Males (***N*****=** 300)**										
WO	4–11	12–13	14–20	12.35	3.32	0.27	0.28	0.20	−0.26	0.39
AG	4–7	8–9	10–20	8.52	3.00	0.24	0.78	0.20	0.26	0.39
AW	4–12	13–15	16–20	13.58	2.76	0.22	−0.13	0.20	0.86	0.39
PR	2–5	6	7–10	5.72	1.64	0.13	0.21	0.20	0.71	0.39
PSE	3–10	11–12	13–15	10.95	2.55	0.21	−0.78	0.20	0.88	0.39
**Females (***N*****=** 788)**										
WO	4–12	13–16	17–20	14.03	3.22	0.16	0.03	0.12	−0.79	0.24
AG	4–9	10–12	13–20	10.37	3.41	0.17	0.37	0.12	−0.36	0.24
AW	4–13	14–15	16–20	14.47	2.55	0.08	0.32	0.12	−0.25	0.24
PR	2–6	7	8–10	6.60	1.69	0.08	0.32	0.12	−0.25	0.24
PSE	3–10	11–12	13–15	10.56	2.55	0.13	−0.49	0.12	−0.04	0.24

*WO, worry; AG, agitation; AW, awareness; PR, prosociality; PSE, perceived self-efficacy; SD, standard deviation; SE, standard error; SK,skewenes; KU, kurtosis. The score ranges (low, medium, high) correspond to the percentiles below 25, 25–75, above 75*.

## Discussion

The analyses carried out led to the definition of a scale composed of a total of 18 items that converge separately on four factors. The first factor measures the person's worry about the estimated negative consequences of the current situation: i.e., basic necessities, money availability, extensive consequences of the crisis, questioning of future projects. The aspect of material assessment and the concern for the objective change that one's life is undergoing due to the current emergency situation prevails. The convergent validity analysis indicated the significant association with the two components (somatic-affective and cognitive) of the depression scale. It can therefore be said that the person with a high score on the Worry scale could present mood declines, dysphoria, general dissatisfaction with present life conditions and the results achieved, melancholy and nostalgia for past events, sadness, pessimism, low self-esteem, propensity to self-criticism, loss of energy and motivation, difficulty in concentration, fatigue, and sleep disorders. Excessive and persistent worry can represent a real block for the person, who cannot functionally channel his/her energies to deal with and solve the tasks of his/her present condition, and may activate symptoms of anxiety and PTSD. Depressed people presumably assess such situations as more stressful and less controllable than non-depressed people. As shown in several studies, they exert less active influence on controllable adverse stressors and have a greater passivity (hesitation and resignation) and a greater tendency to escape (avoidance, withdrawal, escape) and tend to be more self-critical by blaming and belittling themselves (Ginexi et al., 2000; Tracy et al., 2011; Wilson-Genderson et al., 2018; Mamun et al., 2019; Lei et al., 2020).

The second factor of the ERPAS measures the person's agitation reaction (i.e., anxiety, stress, fears, irritability). The simultaneous occurrence of these last four reactions is listed in the literature as symptoms of BTSD (DSM-5, APA). The agitation component emphasizes the bodily (also somatization) and behavioral response to the stressful event. Because of stress, the person can activate totally or partially unconscious responses that impact on their well-being and overall psychological balance. Most of the time the individual is unable to control his/her tensions, fears, sense of frustration and loss, reacting either with manifestations of impulsivity and excessive irritability or showing a defensive closure in themselves and the inability to manage their usual social relationships with naturalness. According to the DSM-5, the defensive mode is constantly activated, resulting in a physiological state of hyper-arousal that does not end naturally. The person develops a sort of hypersensitivity to potential danger signals, which leads him/her to be constantly on the alert, to respond in an explosive and angry manner even in the absence of provocation and to live in a state of hypervigilance and tension that interferes with the ability to calm down or fall asleep (Criterion E: symptoms of hyperactivation. DSM-5, 2013). The convergent validity analysis reported in our study a strong positive correlation with measures of depression, but also strong negative correlation with Self-Efficacy. This shows that stiffening and closure also leads to a loss of self-confidence and the ability to cope with the difficulties and problems that have arisen. Several prior studies have found associations between disaster-related losses and the severity and persistence of both PTSD and depressive symptoms (Goenjian et al., 2000; Armenian et al., 2002; Galea et al., 2002; Miguel-Tobal et al., 2006; Tracy et al., 2011).

The third factor measures the person's awareness response following emergency circumstances. This is identified through the observation in the person of learning behaviors, focusing on the present, adjustment to changes, daily purpose identification; planning for the future. L'associazione tra benessere, atteggiamento ottimistico e consapevole con il focus temporale presente è stato riportato in Diotaiuti et al. (2021a). The Awareness response is adaptation-oriented: the person becomes aware of the situation, begins to think about how to behave and adapt in this new condition, identifies a daily goal, begins to plan future goals to commensurate with the new situation, and recognizes and is aware of his/her emotions. Our results showed, in accordance with the literature (Caldwell and Hayes, 2016; Lackner and Fresco, 2016; Akinola et al., 2017; Sendzik et al., 2017), a negative association with depressive components and a positive association with Self-Efficacy. Following Saccinto et al. (2013), active and conscious behaviors can reduce post-traumatic stress symptoms, and people who feel more self-effective during the emergency situation have fewer symptoms in the post-event period. Self-efficacy is a protective factor that reduces DPSD symptoms and predicts recovery in victims of natural and man-made disasters (Benight and Harper, 2002; Benight and Bandura, 2004).

The fourth factor measures the response of Prosociality (i.e., solidarity, making oneself available to others, aiding others in need, instilling hope in those around; empathizing with others). It constitutes an individual's spontaneous openness toward others when an emergency situation arises; this overcomes the response of fear by activating one's own internal resources and channeling them into concrete and immediate actions toward the community. As can be seen from the associations highlighted by the convergent analysis, this reactive disposition entails a greater sense of self-efficacy and above all a strong negative correlation with the cognitive dimension of depression. The more the person makes himself available to others, helping them concretely, the more he feels useful and increases his perception of self-efficacy. The active momentum limits the opportunities for remorse and self-criticism and the sense of stalemate and blockage (Klein and Epley, 2014; Futamura, 2018; Leder et al., 2020; Shi et al., 2020). As pointed out by Meng and Meng (2020), encouraging prosocial behavior is also an effective way to improve mindfulness in highly ruminative individuals.

The fifth factor measures perceived self-efficacy in an emergency situation (i.e., feeling able to face unexpected events, relying on one's own abilities in emergencies, being able to remain calm in facing difficulties). This is a very delicate aspect in the evaluation of emergency response because the occurrence of threat and damage significantly affects the psycho-physical balance of the person and interferes with the ability to feel able to face difficulties (Weber and Schulenberg, 2019; Diotaiuti et al., 2021b). Self-efficacy has been associated in several studies with improved behavioral response and recovery in the face of various threats and traumas (Tang and Wong, 2003; Benight et al., 2008; Cieslak et al., 2008, 2009; Hirschel and Schulenberg, 2009; Bults et al., 2011; Williams et al., 2015; Adams et al., 2019).

The measurement invariance analysis with respect to gender revealed important aspects related to potential gender differences in the person's experience of the emergency and recovery potential. Comparison of the values of the latent averages in the factors comprising the ERPAS instrument showed that among the participants in our study, women on the one hand reported values indicating a response of greater tension and worry, yet on the other hand showed higher values than men on two important factors for recovery, namely situational awareness and perceived self-efficacy.

These findings are important because they fit within the current debate on interpreting gender differences in coping with extreme stressful emotions and in disaster preparedness and recovery from traumatic situations. The importance of gender in the response to and recovery from disasters has been recognized as a priority by many humanitarian organizations (as indicated by Moreno and Shaw, 2018). The here reported values of greater agitation and worry in females are consistent with other studies such as that of Ziabari and Treur (2018) in which particular differences in rumination and decision making emerged: extreme emotion causes rumination in females more than in males, that generally deal with such a situation by “fighting or flying,” which means facing an extreme emotion or running away from it. According to Ziabari and Treur (2018), females generally have their own policy called “tend-and-befriend,” which means they consult the tough situation with others to find a better result in facing with the extreme emotion and acute stress. It is likely that their collaborative and constructive attitudes are associated with a joint search for greater awareness and evaluation of the effectiveness of their own and their reference group's abilities in coping with and being resilient to events. However, while disaster research has certainly explored women's vulnerability, women's resilience is less well documented. These observations are now widely reported in more recent studies of women's leadership in disasters (Wisner et al., 2016; Gaillard et al., 2017; Clissold et al., 2020). They advocate for the recognition of women's needs as well as their strengths and assets. Resilience emphasizes that women are not merely passive recipients of aid; they are active agents (Gaillard et al., 2015); and studies show the crucial role played by women in caretaking, communicating risks, organizing communal activities, and building new partnerships (see Dhungel and Ojha, 2012; Shah, 2012).

The results of our study, relating to the administrations carried out in Italy during the period of spread of contagions and deaths due to the corona virus in 2020, indicate that in women the greater agitation and concern was positively compensated by awareness and perception of self-efficacy, while substantially no difference compared to males appeared in the values of prosociality. This may indicate, in the face of the fear for the situation experienced, an attitude not paralyzed, stiffened, and depressed, but rather aware, constructive and proactive, open to the sharing of information and functional actions to overcome difficulties.

The measurement of models of emotional, cognitive, and behavioral response to emergency situations is an important aspect for the assessment of the ability to adapt to drastic change, resource limitation, emotional pressure, and loss. The identification of a dominant model of closure is useful for predicting episodes and conditions of high levels of stress, anxiety, depression, and worry. On the other hand, a prevalent open and supportive response model appears more functional to adaptation and predicts a greater sense of self-efficacy in dealing with the difficulty, better organizational and operational skills in responding to the needs that have arisen and the maintenance of a mental scenario aimed at planning. Within the ERPAS scale the evaluation of individual awareness in the situation is a key factor to infer on the level of emotional regulation, on the learning ability of the person in the given situation and on the predisposition to activate and mobilize one's own internal resources.

The ERPAS tool prefigure an application during the assessment in multiple emergency contexts (e.g., earthquakes, floods, pandemics, terrorist attacks, war events, major accidents, major fires). Since the scale reveals responses and adaptations to emergency situations, it may be useful to make several administrations using the same scale in order to also verify the effect of the care actions taken by psychosocial emergency teams and to prevent the development of subsequent mental disorders (depression, PTSD, panic attacks, adaptation disorders). The scale should be administered at an early stage of psychological rescue for an initial assessment of responses to the impact of the event, also in order to prioritize the cases for the referral. Therefore, high scores in Worry and Agitation, and low scores in Awareness, Prosociality, Perceived Self-Efficacy may need a referral, after the necessary interventions such as PFA (Psychological First Aid). Higher scores on Awareness, Prosociality, Perceived Self-Efficacy, and low scores in Worry and Agitation are considered as more resilient.

If the instrument reveals a closing response (excessive concern, state of anxiety, high levels of psychological stress) at the end of the psychological first aid, it can be envisaged to send the person to specialized care and treatment services (IASC, 2007). A few weeks later, the administration can be repeated to ascertain the actual change/adaptation in the situation, specifically assessing the level of awareness, the activation of resources, the perception of self-efficacy (Snider et al., 2011). It would still be desirable for practitioners not to use ERPAS as a screening tool to prioritize the people to access the PFA service. Emergency Response and Psychological Adjustment Scale only provides a general outline of their distress and resources to cope, nevertheless, the situation of a person may change quickly in the aftermath of a disaster or emergency. It would be nice to limit the potential misuse of ERPAS. Psychological First Aid should be accessible for all irrespective of the ERPAS scores, however, the practitioners can monitor the situation (progress/deterioration) of the person by using pre-post ERPAS scores as a component of their PFA service.

## Study Limitations

In terms of limitations, the present study based the validation on the involvement of a sample affected by the Covid-19 pandemic and the administration took place during the most intense lockdown period in Italy. A further verification of the validity of the instrument should therefore imply the extension of the study to samples of individuals affected by different forms of emergency and catastrophe. Additional research involving breadth of content may provide a greater increase in the validity of the ERPAS. This study was also limited by the reliance on an on-line survey method of evaluation and self-report measurement. Likewise, additional methods of assessment, such as interviews to evaluate the scope of avoidance and the inclusion of new safety behaviors, may reveal additional indicators of cognitive emotional/ and behavioral responses to emergencies. Further research should through a test-retest method prove the reliability/sensitivity of the instrument in measuring affective, emotional, cognitive changes related to the coping in the person of the emergency situation.

## Conclusion

This validation study of the *ERPAS* has shown that this version is a reliable measurement for assessing people's modes of personal response (cognitive, emotional, behavioral) in emergency contexts. The convergent validity assessment confirmed predictive indications with variables such as cognitive and somatic depression and perceived general self-efficacy. The analyses also showed a strong invariance across gender. The availability of this new tool is also intended to be a stimulus to encourage new comparative studies to test the adequacy of the ERPAS model on specific samples of the population and in relation to different types of emergencies.

## Data Availability Statement

The raw data supporting the conclusions of this article will be made available by the authors, without undue reservation.

## Ethics Statement

The studies involving human participants were reviewed and approved by Institutional Review Board of the University of Cassino and Southern Lazio. The participants provided their written informed consent to participate in this study.

## Author Contributions

PD, GV, and SM designed the study, analyzed the data, and discussed the results. PD and GV drafted the manuscript. SM and GV revised the manuscript. Finally, the authors have agreed to be accountable for all aspects of the manuscript in ensuring that questions related to the accuracy or integrity of any part of it are appropriately investigated and resolved. All authors read and approved the final manuscript.

## Conflict of Interest

The authors declare that the research was conducted in the absence of any commercial or financial relationships that could be construed as a potential conflict of interest.

## Publisher's Note

All claims expressed in this article are solely those of the authors and do not necessarily represent those of their affiliated organizations, or those of the publisher, the editors and the reviewers. Any product that may be evaluated in this article, or claim that may be made by its manufacturer, is not guaranteed or endorsed by the publisher.
